# Compulsory but Unpaid Medical Services

**Published:** 1902-10-11

**Authors:** 


### EDITOR'S LETTER-BOX.

**[Our Correspondents are reminded that prolixity is a great bar to publication, and that brevity of style and conciseness of statement greatly facilitate early insertion.]**

### COMPULSORY BUT UNPAID MEDICAL SERVICES.

J. R. Williamson writes:—

With reference to the able and significant leading article in The Hospital of 20th inst. on "Compulsory but unpaid professional services," will you kindly permit me to point out that the draft Bill for Amendment of the Law of Burial drawn up by the London Association for the Prevention of Premature Burial, for presentation to Parliament, provides for the due remuneration of medical officers and others of the medical profession? Section 6 is as follows:—"Medical officers and other medical men performing any duty under this Act shall be entitled to such fees as may be allowed by rules made under the authority of the Local Government Board; and such fees shall be paid out of the rates of the district. Subject to any such rules, the inspection and certification may be charged for as medical attendance against the estate of the alleged deceased person or the party or parties applying for the same."

A terrible case of burial alive has recently been reported from Buenos Ayres which illustrates the awful results of hasty medical diagnosis and disposal of the apparent dead. It appears that during a grand celebration of the 18th birthday of Mdlle. Cambarceres, a descendant of the famous French general of that name, and a member of one of the leading families in the Argentine capital, the unfortunate young lady fell to the ground apparently lifeless. In accordance with municipal law she was buried within 24 hours. A few days later the story got about that Mdlle. Cambarceres had been poisoned, and the body was exhumed. When the coffin was opened it was discovered that the veil which had been placed over the supposed corpse was torn, and that her face was covered with scratches. This is a peril to which we are all more or less exposed, because no precautions whatever are taken to avert it, and since exhumation does not occur once in 100,000 interments, there must be thousands of undiscovered cases of premature burial for one brought to light. It will be said that as burials do not take place in England so soon after death, the danger of similar tragedies is very rare, and people need not alarm themselves about it. This view entirely overlooks the wellascertained fact that trance and other forms of suspended animation often continue for several days, and sometimes for weeks. All medical authorities are now practically agreed that the only certainty of dissolution is putrefactive decomposition, which in this country is not often waited for before the body is embalmed, buried, or cremated. So much impressed with the danger of live sepulture was the late Sir B. W. Richardson, M.D., F.R.S., that he prescribed a number of signs and proofs of death, but, unfortunately for the public safety, they are rarely if ever employed in general medical practice. As soon as a person appears to die he is certified as actually dead* by the attendant doctor—often on the unsupported testimony of friends, and without seeing and examining the body—and immediate instructions are given to the undertaker to prepare for the funeral.

[* This is hardly a correct statement. The Act requires that if the deceased has been "attended during his last illness by a registered medical practitioner, that practitioner shall sign and give to some person required to give information concerning the death a certificate stating to the best of his knowledge and belief the cause of death." The certificate does not certify to the fact of death. As to the fact of death having taken place, the registrar has, apparently, to accept the statement of the "person required to give information concerning the death." We do not agree with our correspondent as to the danger of premature burial in this country, but we have no doubt about the extremely unsatisfactory condition of existing arrangements in regard to registration and burial. That there exists no official provision for ascertaining either the fact of death or the identity of the person who is said to be dead, and that except in cases attended by registered medical practitioners the cause of death is left to be guessed at by any old woman who happens to be present at the death, is a highly discreditable state of affairs, which one might expect to attract the attention of our law givers. Modern legislators, however, wait for a mandate.—Ed. The Hospital.]

